# Serum levels of PTEN and progranulin as potential diagnostic and prognostic biomarkers for severe pneumonia in the elderly

**DOI:** 10.7717/peerj.20956

**Published:** 2026-03-24

**Authors:** Yi Ge, Wei Lu, Jinlong Liu, Huan Liang, Linlin Xu

**Affiliations:** Department of Geriatrics, Shuyang Nanguan Hospital, Suqian, Jiangsu, China

**Keywords:** Severe pneumonia, PTEN, Diagnosis, Prognostic assessment, Progranulin

## Abstract

**Background:**

Severe pneumonia presents a critical challenge clinically, especially for elderly patients. This study investigates the diagnostic and prognostic potential of serum Phosphatase and Tensin Homolog Deleted on Chromosome Ten (PTEN) and Progranulin in this population.

**Objectives:**

To evaluate the effectiveness of phosphatase and tensin homolog deleted on chromosome ten (PTEN) and Progranulin as diagnostic and prognostic biomarkers for differentiating severe pneumonia from common pneumonia in elderly patients, so as to enhance clinical practice.

**Methods:**

This comparative study was conducted on 90 elderly patients with severe pneumonia (Observation Group) and 90 elderly patients with common pneumonia (Control Group). Based on the survival outcome 28 days post-admission, the Observation Group was further divided into the Survival Group (*n* = 51) and the Deceased Group (*n* = 39). This study continued to measure and compare the white blood cell counts, albumin, procalcitonin, C-reactive protein (CRP), as well as serum levels of PTEN and Progranulin. Spearman correlation analysis was used to assess correlations between PTEN and Progranulin levels. In addition, the diagnostic efficacy of PTEN and Progranulin was assessed using receiver operating characteristic (ROC) curves. Besides, univariate and multivariate logistic regression analyses were employed to determine mortality-related factors.

**Results:**

Patients with severe pneumonia were measured with significantly higher white blood cells, CRP, procalcitonin, PTEN, and Progranulin levels (all *P* < 0.05), but lower albumin levels (all *P* < 0.05). PTEN exhibited a moderately positive correlation with Progranulin. The combined use of PTEN and Progranulin yielded a higher area under the curve (AUC) of 0.887. Additionally, elevated CRP and Progranulin levels were linked to worse outcomes, and Progranulin was a novel independent risk factor for patient death.

**Conclusion:**

Serum PTEN and Progranulin are promising diagnostic biomarkers for severe pneumonia in elderly patients, with Progranulin showing prognostic value in particular.

## Introduction

Severe pneumonia has been recognized to be a significant challenge in clinical settings given its pathological feature as a prevalent and potentially life-threatening respiratory illness (Zhu et al., 2023). It has a demographic landscape manifesting as an increasing prevalence among elderly individuals in recent decades, further heightening the complexity of managing such cases (Falcone et al., 2023). Due to compromised lung cell and ciliary defense mechanisms, elderly patients, in particular, face higher vulnerability, culminating in weaker immunity and poorer prognoses compared to younger patients (Ocrospoma & Restrepo, 2024). When encountering severe pneumonia, usually marking a critical juncture, the elderly may experience a loss of autonomy, cognitive decline, exacerbation of existing comorbidities, and even mortality (Martin, Mannino & Moss, 2006). Given these challenges, the identification of precise diagnostic and prognostic biomarkers is paramount to the management of severe pneumonia in the elderly effectively.

Phosphatase and tensin homolog deleted on chromosome ten (PTEN) is a typical tumor suppressor that functions as a lipid and protein phosphatase, which is pivotal for inhibiting the Phosphatidylinositol 3-kinase (PI3K)/Akt pathway, thereby regulating cell cycle (Looker et al., 2022). PTEN stands out as one of the most significant biomarkers involved in governing various processes linked to the onset and progression of diverse diseases, notably cancer (Álvarez-Garcia et al., 2019; Schabbauer et al., 2010). This multifaceted protein has also been found to be essential in lung diseases. Recently, Cai et al. (2022) summarized the role of PTEN expression and regulation in a series of airway pathological conditions (*e.g.*, asthma/allergic airway inflammation, pulmonary arterial hypertension (PAH), chronic obstructive pulmonary disease (COPD), idiopathic pulmonary fibrosis (IPF), and other acute lung injuries (ALI)). Moreover, some studies have also proposed the possibility of targeting PTEN in lung disorder treatment. Specifically, PTEN expression can be enhanced by the SNHG5-miR-132 signaling axis, which can lower the expression of inflammatory factors (IL-1β, IL-6 and TNF-α), thereby suppressing the progression of COPD. Meanwhile, PTEN inhibition can weaken the inhibitory effect of SNHG5 upregulation on the progression of COPD (Shen et al., 2020). Huang et al. (2025) discovered that miR-142-3p could regulate airway inflammation through the PTEN/AKT signaling pathway to alleviate symptoms related to asthma. Yang et al. (2023) also found that the role of silencing EIF3A to reduce PASMC proliferation in improving PAH depended on the modulation of the HDAC1-mediated PTEN/PI3K/AKT pathway. Simultaneously, Zhu et al. (2025) also found the involvement of the classic signaling pathway of PTEN/PI3K/Akt in improving PAH. Some other researchers also believed that activating PTEN can inactivate the PI3K/AKT/mTOR/NF-κB signaling pathway to hinder the senescence of AECs and further inhibit IPF progression (Li et al., 2024; Zhang et al., 2024b).

Progranulin is a secretory glycoprotein comprising 593 amino acid residues, serving as both an actor and a regulator in host immune responses and inflammation (Liu et al., 2024). Progranulin is a versatile factor that usually acts as an autocrine growth factor, anti-inflammatory agent, or adipokine, depending on the specific target tissue (Meda et al., 2023). With a wide spectrum of pathophysiological mechanisms, Progranulin can participate in tissue damage repair, tumorigenesis, neurodegeneration, COPD, and bronchial asthma (Liu et al., 2024; Na, Heng & Hao, 2023). The expression and regulation of Progranulin have been reported to exert intimate associations with the progression of lung diseases (Chen et al., 2020; Lu et al., 2021; Luo et al., 2020; Tóth et al., 2023; Xie et al., 2021; Zhang et al., 2024a). Zhao et al. (2024) discovered that by reducing the IL-6 and Tgf-β1/Smad signaling pathways, targeting Progranulin could alleviate pulmonary inflammation and fibrosis. Furthermore, Progranulin can suppress B cell activation and IgE production through the IFITM3-STAT1 signaling pathway, providing a new strategy for targeted therapy of bronchial asthma (Zhang et al., 2024a). Progranulin can also remarkably restrain cellular inflammatory responses through the MAPK pathway, increase lung permeability, and reduce inflammatory cytokine expression in BALF and serum, thereby alleviating ALI (Lu et al., 2021). Similarly, Progranulin can independently predict chronic COPD, which may also mediate its pathogenesis by interfering with the inflammatory response (Chen et al., 2018).

In general, the elderly individuals are more prone to illness than middle-aged and young people, due to the gradual aging of their various organs and the gradual decline of their immunity. As evidenced previously, the mTOR pathway is overactivated during aging, and targeting PTEN can inhibit autophagy and promote inflammation, driving the decline of immune cell function (*e.g.*, T cell exhaustion), and exacerbating chronic inflammation and immunosenescence (Fu et al., 2025). Furthermore, the deficiency of PTEN can induce metabolic geriatric diseases. Abnormal function of PTEN can trigger dysfunction of the mitochondrial autophagy, causing reactive oxygen species accumulation, chronic inflammation and energy metabolism disorders, and accelerating the progression of diabetic nephropathy, cardiomyopathy, *etc.* (Hu et al., 2025). Meanwhile, there is a gradual accumulation of Progranulin during physiological aging. Its ability to inhibit the IGF-1/Akt signaling pathway weakens the body’s regulatory capacity for metabolism and growth, thereby exacerbating the aging (*e.g.*, tissue degeneration and functional decline) (Jiang et al., 2022).

Currently, we know little about the relationship of serum PTEN and Progranulin levels with severe pneumonia in the elderly. Given this context, our study proposed a hypothesis that serum PTEN and Progranulin could serve as effective diagnostic and prognostic biomarkers for severe pneumonia in the elderly, thereby identifying their diagnostic and prognostic values.

## Materials and Methods

### Study subjects

Between April 1, 2023 and March 31, 2025, this study was initiated with the selection of 90 elderly patients with severe pneumonia (Observation Group) admitted to the Geriatrics and Respiratory Departments of Shuyang Nanguan Hospital using the random table method. This group comprised 57 males and 33 females, with a median age of 74.00 (IQR: 67.00–77.25) years (61–86 years). Meanwhile, this study established a Control Group involving 90 elderly patients with common pneumonia treated during the same period. This group consisted of 51 males and 39 females, with the median age of 73.00 (IQR: 68.00–76.25) years (60–88 years). Furthermore, the Observation Group was further divided into two subgroups based on survival outcomes after 28 days of hospitalization. Specifically, the Deceased Group (39 patients), included 27 males and 12 females, with the average age of 72.92 ± 5.84 years (63–85 years); while the Survival Group (51 patients), comprised 30 males and 21 females, with the average age of 73.10 ± 7.58 years (61–86 years). The gender comparison between the Observation Group and the Control Group, as well as between the Deceased Group and the Survival Group was conducted using the chi-square test (categorizing data). The comparison of age and body mass index (BMI) (without normal distribution) between the Observation Group and the Control Group was conducted using a non-parametric test (Mann–Whitney U test); while the age (satisfying normal distribution and homogeneity of variance) comparison between the Deceased Group and the Survival Group used the independent sample *t*-test; and the comparison of BMI (without normal distribution) between the two groups utilized non-parametric tests (Mann–Whitney U test). The baseline characteristics of patients in each group are shown in Tables 1 and 2. There were no statistically significant differences in gender, age and BMI between the Observation Group and the Control Group, as well as between the Deceased Group and the Survival Group (all *P* > 0.05), indicating the comparability between these groups. This study had been officially approved by the Ethics Committee of Shuyang Nanguan Hospital (Approval No.: 20230327018), with written informed consent obtained from the enrolled subjects (or their guardians). We confirmed that we had correctly used the STARD guidelines (Cohen et al., 2016).

**Table 1:** Basic information of patients in control group and observation group.

**Indicator**	**Control group**	**Observation group**		**Z/x2**	***P***
Number of cases (n)	90	90		–	–
Gender^a^	–	–		–	–
Male	51	57		0.833	0.361
Female	39	33			
Age (years)^b^	73.00 (IQR: 68.00–76.25)	74.00 (IQR: 67.00–77.25)		0.029	0.977
BMI^b^	22.00 (IQR:18.00–24.00)	20.00 (IQR:18.00–22.25)		1.881	0.060

**Notes.**

a indicates the chi-square test.

b indicates the Mann–Whitney U test.

**Table 2:** Basic information of patients in deceased group and survival group.

**Indicator**	**Survival** ** group**	**Deceased** ** group**			**Z/T/x2**	***P***
Number of cases (n)	51		39		–	–
Gender^a^	–		–		–	–
Male	30		27	1.031	0.310
Female	21		12		
Age (years)^b^	73.10 ± 7.58		72.92 ± 5.84	0.120		0.905
BMI^c^	20.00 (IQR:18.00–22.00)		21.00 (IQR:19.00–23.00)	1.007		0.314

**Notes.**

a indicates the chi-square test.

b indicates the independent sample *t*-test.

c indicates the Mann–Whitney U test.

### Inclusion and exclusion criteria

Inclusion criteria: (1) meeting the diagnostic criteria for severe pneumonia (Observation Group) and common pneumonia (Control Group) in relevant guidelines (Baek et al., 2020), respectively; (2) aged at least 60 years. (3) with clinical data; and (4) confirmed to have common pneumonia or severe pneumonia through a comprehensive diagnosis including imaging, blood tests, and clinical signs.

Exclusion criteria: (1) combined with severe renal insufficiency or cardiac dysfunction; (2) with tumors or immune system diseases; (3) with other lung diseases, such as COPD and interstitial lung diseases, *etc.* (4) with other underlying diseases, such as diabetes, hypertension and hyperlipidemia, *etc.* (5) with long-term use of hormones; (6) with mental disorders and poor compliance; and (7) with coagulation disorders.

### Detection methods

Both groups of patients were subjected to routine indicator tests, including the measurement of white blood cell count, albumin, procalcitonin, and C-reactive protein (CRP) levels, serving as secondary variables. Simultaneously, the serum levels of PTEN and Progranulin, two primary variables, were determined using the enzyme-linked immunosorbent assay (ELISA) kits (JL13559-96T and JL48587-96T, Shanghai Jianglai Biotechnology Co., Ltd., Shanghai, China) as instructed strictly.

### Comparison of various medical indicators levels between control group and observation group

In terms of the comparison of quantitative data between the Control Group and the Observation Group, the initial step was the determination of normal distribution using the Kolmogorov–Smirnov test. Consequently, a non-parametric test (Mann–Whitney U test) would be adopted directly when the normal distribution was not satisfied; while data distributed normally were further analyzed by the homogeneity of variance test (Levene test). In the context of homogeneous and non-homogeneous variance of the two groups, relevant data would be assessed by the standard independent sample *t*-test or the Welch corrected *t*-test, respectively. Specifically, white blood cells, albumin, CRP, Procalcitonin, PTEN and Progranulin were compared between the Control Group and the Observation Group. Additionally, the categorical data were analyzed using chi square test.

As for correlation analysis of PTEN and Progranulin within the Observation Group, the normality test (Kolmogorov–Smirnov test) was conducted firstly. Subsequently, the Pearson or the Spearman correlation analysis was selected for data satisfying normal distribution and liner relationship, or those not conforming to normal distribution.

### Diagnostic and prognostic monitoring value of PTEN and Progranulin in elderly patients with severe pneumonia

In the evaluation of the diagnostic value of PTEN and Progranulin for severe pneumonia in the elderly, the receiver operating characteristic (ROC) curve was plotted and analyzed correspondingly. The area under the curve (AUC) values between 0.5−0.7, 0.7−0.8, 0.8−0.9, and  > 0.9 would indicate poor, moderate, goody, and excellent predictive performance, respectively. The Youden Index (sensitivity + specificity −1) was employed to determine the optimal threshold, with the sensitivity and specificity corresponding to that point recorded at the same time.

In the assessment of the prognostic monitoring value, a Survival Group and a Deceased Group were constructed based on the survival outcomes of the Observation Group as described previously. Then, all indicators were subjected to traditional univariate analysis for initial screening of indicators. Indicators with statistical differences (*P* < 0.05) were included in the multivariate regression analysis. Finally, a logistic regression was used to analyze the prognostic factors affecting the death of elderly patients with severe pneumonia.

### Statistical analysis

The analysis for this study was carried out using Statistical Product and Service Solutions (SPSS) software version 22.0. The independent sample *t*-test was used to evaluate the differences for quantitative data meeting normality and homogeneity of variance, otherwise alternative methods were used; while categorical data were analyzed using Chi-squared tests. The correlation between PTEN and Progranulin expressions was investigated using Pearson or Spearman correlation analysis. ROC curves were utilized to assess the diagnostic efficacy of PTEN and Progranulin for severe pneumonia in the elderly. Factors influencing mortality were examined through univariate and multivariate logistic regression analyses. A *P*-value of  < 0.05 was considered statistically significant in this study.

## Results

### Comparison of peripheral blood inflammatory markers

The white blood cells, albumin, CRP and Procalcitonin in the Control Group and the Observation Group did not simultaneously satisfy the normal distribution. Therefore, the Mann–Whitney U test was used for statistical analysis. In comparison to the Control Group, the Observation Group displayed higher levels of white blood cells (11.00 (IQR: 9.00–14.00) *vs.* 15.00 (IQR: 12.00–18.00)), CRP (41.50 (IQR: 28.00–58.00) mg/L *vs.* 78.00 (IQR: 59.50–88.00) mg/L), and Procalcitonin (0.53 (IQR: 0.45−0.72) ng/L *vs.* 0.83 (IQR: 0.65−0.88) ng/L), with statistical significance (all *P* < 0.001). Conversely, the Observation Group exhibited lower albumin levels (35.00 (IQR: 32.00–38.00) g/L *vs.* 27.00 (IQR: 24.00–32.00) g/L) (*P* < 0.001) (Table 3).

**Table 3:** Comparison of peripheral blood inflammatory marker levels.

**Indicator**	**Control group**	**Observation group**	***Z***	***P***
Number of cases	90	90	–	–
White blood cells (×10^9^)^c^	11.00 (IQR:9.00–14.00)	15.00 (IQR:12.00–18.00)	6.483	<0.001
Albumin (g/L)^c^	35.00 (IQR:32.00–38.00)	27.00 (IQR:24.00–32.00)	−8.262	<0.001
CRP (mg/L)^c^	41.50 (IQR:28.00–58.00)	78.00 (IQR:59.50–88.00)	8.152	<0.001
Procalcitonin (ug/L)^c^	0.53 (IQR:0.45–0.72)	0.83 (IQR:0.65–0.88)	7.252	<0.001

**Notes.**

c indicates the Mann–Whitney U test.

### Serum PTEN and progranulin levels

Given the inconsistent normal distribution of PTEN and Progranulin, the Mann–Whitney U test was used for statistical analysis. The serum levels of PTEN were notably higher in the Observation Group at 3.40 (IQR: 2.70−3.60) ng/mL compared to 2.50 (IQR: 1.58−2.80) ng/mL in the Control Group (*P* < 0.001). Similarly, Progranulin levels were obviously higher in the Observation Group, registering at 78.50 (IQR: 71.50–89.00) ng/mL in contrast to 65.00 (IQR: 60.38–71.75) ng/mL in the Control Group (*P* < 0.001) (Table 4).

**Table 4:** Serum levels of PTEN and progranulin patients and controls.

**Indicator**	**Control group**	**Observation group**	***Z***	***P***
Number of cases	90	90	–	–
PTEN (ng/mL)^c^	2.50 (IQR:1.58–2.80)	3.40 (IQR:2.70–3.60)	6.688	<0.001
Progranulin (ng/mL)^c^	65.00 (IQR:60.38–71.75)	78.50 (IQR:71.50–89.00)	7.780	<0.001

**Notes.**

c indicates the Mann–Whitney U test.

### Correlation between serum PTEN and progranulin levels for severe pneumonia in the elderly

The Spearman correlation method was employed for analyzing the correlation between serum PTEN and Progranulin levels in the Observation Group considering their non-normal distribution. The results showed that PTEN and Progranulin were moderately positively correlated (*r* = 0.432, *P* < 0.001) (Table 5).

**Table 5:** Correlation Bbetween serum levels of PTEN and Progranulin for severe pneumonia in the elderly.

**Indicator**	**Kolmogorov–Smirnov test**		**Spearman correlation analysis**	
	**D**	***P***	***r***	***P***
PTEN	0.274	<0.001	0.432	<0.001
Progranulin	0.125	0.001		

### Combined diagnostic value of serum PTEN and progranulin for severe pneumonia in the elderly

**Figure 1:**
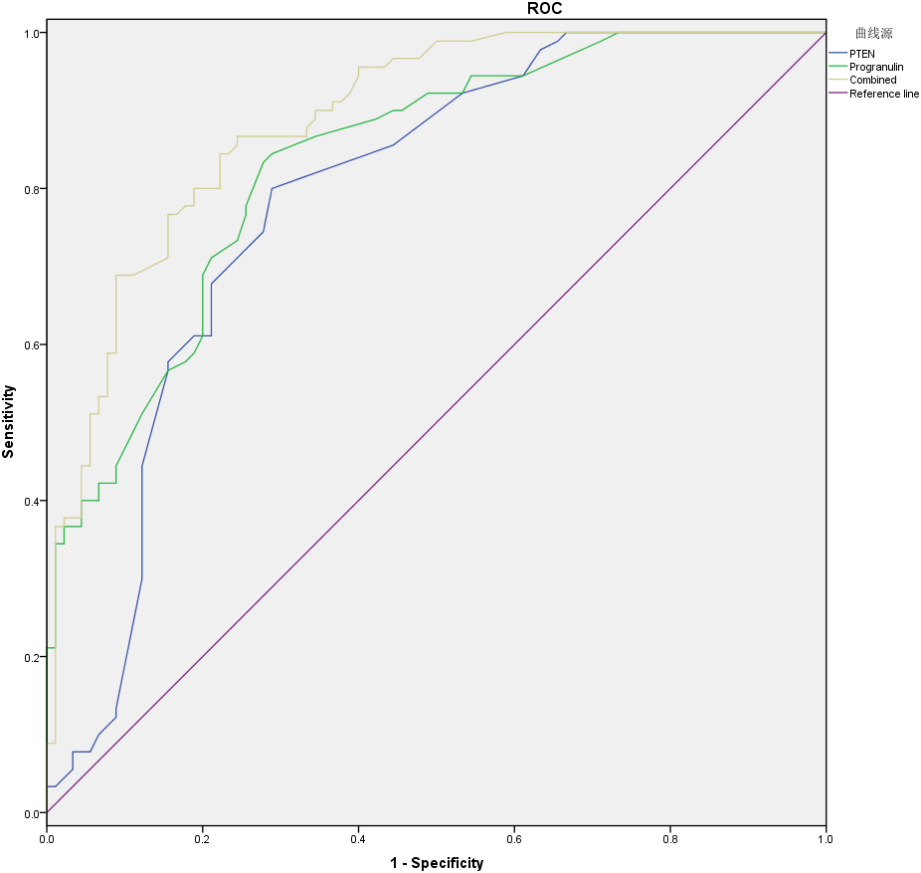
The combined diagnostic value of serum PTEN and Progranulin for severe pneumonia in the elderly.

PTEN had a moderate diagnostic value, given its sensitivity of 80.00% and specificity of 71.10%, with a cutoff value of 2.65 and an AUC of 0.788. While Progranulin revealed a high diagnostic value, considering its slightly higher sensitivity of 83.30% and specificity of 72.20%, along with a cutoff value of 68.75 and an AUC of 0.836. Noticeably, the combination of PTEN and Progranulin indicated an even higher diagnostic value, yielding a sensitivity of 86.70% and specificity of 75.60%, with an AUC of 0.887 (Fig. 1) (Table 6).

**Table 6:** Combined diagnostic value of serum PTEN and progranulin for severe pneumonia in the elderly.

**Indicator**	**Cut-off value**	**Sensitivity (%)**	**Specificity (%)**	**AUC**	**95% CI**	***P*-value**
**PTEN**	2.65	80.00	71.10	0.788	(0.720, 0.856)	<0.001
**Progranulin**	68.75	83.30	72.20	0.836	(0.778, 0.893)	<0.001
**Combined**	–	86.70	75.60	0.887	(0.841, 0.934)	<0.001

### Univariate analysis of prognostic factors for severe pneumonia in the elderly

Mann–Whitney U test was used for statistical analysis of white blood cells, albumin, procalcitonin, PTEN and Progranulin in the Deceased Group and the Survival Group given their non-simultaneous satisfaction of the normal distribution. While the standard independent sample *t*-test was used for statistical analysis of CRP in both the Deceased Group and the Survival Group as it simultaneously met the normal distribution and homogeneity of variance. There was no statistically significant difference in white blood cells (15.00 (IQR: 13.00–18.00) *vs* 14.00 (IQR: 12.00–17.00)), albumin (27.00 (IQR: 24.00–30.00) *vs* 28.00 (IQR: 24.00–33.00)) and procalcitonin (0.83 (IQR: 0.73−0.98) *vs* 0.82 (IQR: 0.65−0.86)) between groups (all *P* > 0.05). However, there were significant inter-group differences in CRP (85.85 ± 14.51 *vs* 63.57 ± 17.90), PTEN (3.50 (IQR:3.40−3.60) *vs* 2.90 (IQR: 2.60−3.50)) and Progranulin (89.00 (IQR: 86.50–93.00) *vs* 74.50 (IQR: 68.00–79.00)) (all *P* < 0.001) (Table 7).

**Table 7:** Univariate analysis of prognostic factors for severe pneumonia in the elderly.

**Indicator**		**Deceased group**	**Survival** **group**	**Z/T/x2**	***P***
Number of cases (n)		39	51		
White blood cells (×10^9^)^c^		15.00 (IQR:13.00–18.00)	14.00 (IQR:12.00–17.00)	0.479	0.632
Albumin (g/L)^c^		27.00 (IQR:24.00–30.00)	28.00 (IQR:24.00–33.00)	1.205	0.228
CRP (mg/L)^b^		85.85 ± 14.51	63.57 ± 17.90	6.339	<0.001
Procalcitonin (ug/L)^c^		0.83 (IQR:0.73–0.98)	0.82 (IQR:0.65–0.86)	1.678	0.093
PTEN (ng/mL)^c^		3.50 (IQR:3.40–3.60)	2.90 (IQR:2.60–3.50)	4.421	<0.001
Progranulin (ng/mL)^c^		89.00 (IQR:86.50–93.00)	74.50 (IQR:68.00–79.00)	5.822	<0.001

**Notes.**

b Indicates the independent sample *t*-test.

c Indicates the Mann–Whitney U test.

### Multifactorial logistic regression analysis of prognostic factors for severe pneumonia in the elderly

In this study, multifactorial logistic regression analyses were further performed utilizing the death of patients with severe pneumonia as the dependent variable, and levels of CRP, PTEN, and Progranulin as independent variables. The results indicated that CRP, and Progranulin emerged as predictors of prognosis (both *P* < 0.05), while PTEN did not exhibit significance (*P* > 0.05) (Table 8).

## Discussion

Elderly patients, with multiple comorbidities and weakened immune systems, may usually significant challenges when suffering from severe pneumonia. This complex clinical profile presents significant obstacles to effective treatment (Xu et al., 2019). It remains elusive regarding definitive and highly efficient diagnostic method, despite the employment of various molecular screening techniques currently to monitor the progression of severe pneumonia. Therefore, it is critical to identify accurate and reliable biomarkers for both diagnosis and prognosis.

To date, the roles of PTEN and Progranulin have been documented in the physiopathology of pulmonary diseases. Yanagisawa et al. reported significantly reduced expression of the protein PTEN, which normally regulates the PI3K pathway, in COPD patients, leading to increased PI3K activation and inflammation. Moreover, cigarette smoke-induced stress could decrease PTEN levels, a process that could be mitigated by N-acetyl cysteine. Meanwhile, reduced PTEN levels would enhance the production of proinflammatory cytokines, thereby amplifying inflammation in COPD (Yanagisawa et al., 2017). PTEN also exhibited obviously reduced levels in allergen-induced asthma. This reduction led to increased PI3K activity and elevated levels of inflammatory markers such as IL-4, IL-5, and eosinophil cationic protein. Significantly, the administration of PI3K inhibitors or PTEN cDNA notably decreased bronchial inflammation and airway hyperresponsiveness, indicating its critical role in the development of asthma (Kwak et al., 2003).

**Table 8:** Multivariate logistic regression analysis of prognostic factors for severe pneumonia in the elderly.

**Indicator**	***P* value**	**OR**	**95% CI**
**CRP (mg/L)**	0.001	1.100	(1.041, 1.163)
**PTEN (ng/mL)**	0.061	5.804	(0.924, 36.437)
**Progranulin (ng/mL)**	<0.001	1.193	(1.083, 1.314)

Progranulin plays a detrimental role in the inflammatory response during influenza virus infection. For instance, Progranulin was reported to be up-regulated in both clinical and experimental influenza cases, contributing to severe pulmonary injury and increased mortality. Progranulin-deficient mice would exert protective function against these harmful effects, resulting in lowered immune cell influx, cytokine and chemokine release, as well as alveolar-epithelial barrier permeability. Notably, this protective effect did not compromise viral clearance, suggesting the role of Progranulin in exacerbating lung immunopathology during influenza infection without affecting the ability to clear the virus (Luo et al., 2019). Choi et al. (2020) discovered the stimulating effect of Progranulin in the production of IL-4 and IL-13 in NKT cells, as well as IL-33 and TSLP in airway epithelial cells. The absence of NKT cells would abolish Progranulin-induced Th2 cytokine production. Moreover, allergic inflammation was significantly reduced in Progranulin-deficient mice, which, however, was restored following recombinant Progranulin administered during sensitization. Collectively, by inducing type 2 cytokine production in NKT and airway epithelial cells, macrophage-derived Progranulin is crucial for initiating Th2 immune responses and allergic airway inflammation.

In this study, both PTEN and Progranulin had significantly higher levels in patients with severe pneumonia than those of the controls. Interestingly, erum PTEN correlated positively with Progranulin levels, suggesting a potential synergistic relationship of the two factors in severe pneumonia. Furthermore, according to the evaluation of their diagnostic performance using ROC curve analysis, PTEN demonstrated a sensitivity of 80.00% and a specificity of 71.10%, while Progranulin exhibited slightly higher values. Notably, PTEN combined with Progranulin yielded superior diagnostic accuracy compared to the use of either marker alone, underscoring their potential clinical utility when used in combination. However, the diagnostic value of this combined diagnostic model may be overestimated given the moderate correlation between PTEN and PGRN. Similarly, prior research has revealed that circulating levels of PTEN and Progranulin may serve as valuable complementary biomarkers across various diseases. For example, in oral squamous cell carcinoma, the ratio of circulating miRNA-21/PTEN levels significantly improved the discriminatory power of nucleic acids in distinguishing cases from controls, achieving a specificity of 62.5% and a sensitivity of 84.5% (Ren et al., 2014). Therefore, PTEN and Progranulin, particularly when assessed jointly, may enhance the diagnostic precision in severe pneumonia. While their clinical applicability remains to be further validated in larger, independent cohorts.

Furthermore, the clinical significance of PTEN and Progranulin has been demonstrated in various disease contexts. For instance, PTEN was downregulated in acute pancreatitis, showing promise in identifying secondary infections in these patients when assessed concurrently with IL-8 and ICAM-1 (Yang Xiaojuan et al., 2023). Lu et al. (2021) reported that it could inhibit the phosphorylation of the MAPK pathway to exert a significantly suppressive role in inflammatory responses, thereby influencing macrophage polarization and improving the survival in acute lung injury. Nevertheless, given that our study was designed as a clinical research, we did not delve into the further molecular mechanisms underlying its occurrence. In the future, on the basis of findings in this study, we will continue to explore its molecular mechanisms, such as the PI3K/AKT/mTOR signaling pathway, MAPK signaling pathway, and relevant regulatory factors. This study demonstrated that Progranulin not only functioned as a prognostic marker for severe pneumonia in the elderly, but also served as an independent risk factor. Notably, PTEN did not show significant prognostic value in this context, although Progranulin emerged as an independent risk factor for mortality. At present, the diagnosis of pneumonia mainly relies on the patient’s clinical symptoms, auscultation of the lungs, laboratory tests, and imaging examinations. Specifically, clinical symptoms and auscultation of the lungs are easily confused with other respiratory diseases. Imaging, despite its use as the gold standard, still has limitations such as possible radiation exposure and poor patient compliance. In terms of laboratory testing, white blood cell count and CRP are commonly used but exhibit poor specificity. The microbiological cultivation of sputum is time-consuming, usually 3–5 days. The diagnostic value of PTEN combined with and Progranulin reached 0.887 in this study, achieving good diagnostic performance. Progranulin can also be used for monitoring the prognosis of severe pneumonia in the elderly. From an economic and time-consuming perspective, each ELISA experiment only costs a few tens of yuan on average, and corresponding results can be obtained in as fast as 1 h. Therefore, results of our study may guide further diagnosis and treatment of severe pneumonia in the elderly. Currently, the mortality of pneumonia has an intimate association with some factors, such as pneumonia severity index, CURB-65, CRB-65, A-DROP, SMART-COP, need for ventilation, and comorbidities. Our study supported that Progranulin was an independent risk factor for mortality in this patient population, supplementing the influencing factors for the clinical diagnosis and treatment of pneumonia. Of course, in the future, the current lethal factors of pneumonia should be combined with the conclusions of this study for further in-depth analysis.

It should be acknowledged that this study has several limitations. Firstly, our investigation might be significantly complicated considering the intricate pathophysiology of severe pneumonia and the unique characteristics of the elderly. Most elderly patients with pneumonia may have some complications, including diabetes, hypertension, COPD, *etc.* But these factors were excluded in our initial consideration of inclusion and exclusion criteria, which may limit the clinical practice of the conclusions of this study. Furthermore, the relatively small sample size (*n* = 90) might compromise the statistical power of our analyses and increase the risk of overfitting in the multivariable logistic regression model. It may eventually weaken the precision of the estimated odds ratios and confidence intervals. Moreover, this study did not include a group of younger pneumonia patients for comparison when exploring the potential diagnostic and prognostic value of PTEN and Progranulin in elderly patients with severe pneumonia. Therefore, age-stratified analyses should be scheduled to determine whether PTEN and Progranulin levels differ between younger and older populations. Of course, the expression of PTEN and Progranulin in elderly healthy populations should be obtained as the baseline levels to further clarify whether PTEN and Progranulin are generally elevated in all pneumonia cases. Simultaneously, for critically ill patients, it is critical to select and elucidate medium to long-term outcomes (*e.g.*, 90 days and 180 days mortality rates) and other clinically important endpoints. Finally, our study noticed relatively modest inter-group differences, although serum Progranulin and PTEN levels might be altered in patients with severe pneumonia. In the future, it is necessary to further confirm these findings and evaluate their clinical significance as well as their potential value in differentiating severe pneumonia in the elderly from common pneumonia in the elderly based on expanded sample size. Overall, there is still a long way to go to identify the mainstream method for classic and stable diagnosis and prognostic monitoring of severe pneumonia in the elderly clinically.

## Conclusion

To sum up, serum PTEN and Progranulin are promising diagnostic biomarkers for severe pneumonia in the elderly. In particular, Progranulin emerges as an independent risk factor for mortality in this population. However, it should be acknowledged that there are some inevitable limitations in this study. It highlights that more in-depth research is warranted to fully evaluate the clinical utility of PTEN and Progranulin for severe pneumonia in the elderly.

##  Supplemental Information

10.7717/peerj.20956/supp-1 Supplemental Information 1 Raw data

10.7717/peerj.20956/supp-2 Supplemental Information 2 STARD Checklist

## Additional Information and Declarations

### Data Availability

The following information was supplied regarding data availability: The data is available in the Supplemental Files.
