# Supplementary material for: Serum levels of PTEN and progranulin as potential diagnostic and prognostic biomarkers for severe pneumonia in the elderly
Source: PeerJ. 2026 Mar 24;14:e20956. doi: 10.7717/peerj.20956 (PMC13024236; doi:10.7717/peerj.20956)
Supplement: Supplemental Information 2 [file peerj-14-20956-s002.docx]

# Reporting checklist for diagnostic test accuracy study.

Based on the STARD guidelines.

## Instructions to authors

Complete this checklist by entering the page numbers from your manuscript where readers will find each of the items listed below.

Your article may not currently address all the items on the checklist. Please modify your text to include the missing information. If you are certain that an item does not apply, please write "n/a" and provide a short explanation.

Upload your completed checklist as an extra file when you submit to a journal.

In your methods section, say that you used the STARDreporting guidelines, and cite them as:

Bossuyt PM, Reitsma JB, Bruns DE, Gatsonis CA, Glasziou PP, Irwig L, LijmerJG Moher D, Rennie D, de Vet HCW, Kressel HY, Rifai N, Golub RM, Altman DG, Hooft L, Korevaar DA, Cohen JF, For the STARD Group. STARD 2015: An Updated List of Essential Items for Reporting Diagnostic Accuracy Studies.

|  |  | Reporting Item | Page Number |
| --- | --- | --- | --- |
| **Title or abstract** |  |  |  |
| None | [#1](https://www.goodreports.org/reporting-checklists/stard/info/#1) | Identification as a study of diagnostic accuracy using at least one measure of accuracy (such as sensitivity, specificity, predictive values, or AUC) |  |
| 1 |  |  |  |
| **Abstract** |  |  |  |
| None | [#2](https://www.goodreports.org/reporting-checklists/stard/info/#2) | Structured summary of study design, methods, results, and conclusions (for specific guidance, see STARD for Abstracts https://www.equator-network.org/reporting-guidelines/stard-abstracts/) |  |
| 1 |  |  |  |
| **Introduction** |  |  |  |
| None | [#3](https://www.goodreports.org/reporting-checklists/stard/info/#3) | Scientific and clinical background, including the intended use and clinical role of the index test | 2-3 |
| None | [#4](https://www.goodreports.org/reporting-checklists/stard/info/#4) | Study objectives and hypotheses | 2-3 |
| **Methods** |  |  |  |
| Study design | [#5](https://www.goodreports.org/reporting-checklists/stard/info/#5) | Whether data collection was planned before the index test and reference standard were performed (prospective study) or after (retrospective study) |  |
| 3-6 |  |  |  |
| Participants | [#6](https://www.goodreports.org/reporting-checklists/stard/info/#6) | Eligibility criteria | 3-6 |
| Participants | [#7](https://www.goodreports.org/reporting-checklists/stard/info/#7) | On what basis potentially eligible participants were identified (such as symptoms, results from previous tests, inclusion in registry) |  |
| 3-6 |  |  |  |
| Participants | [#8](https://www.goodreports.org/reporting-checklists/stard/info/#8) | Where and when potentially eligible participants were identified (setting, location and dates) | 3-6 |
| Participants | [#9](https://www.goodreports.org/reporting-checklists/stard/info/#9) | Whether participants formed a consecutive, random or convenience series | 3-6 |
| Test methods | [#10](https://www.goodreports.org/reporting-checklists/stard/info/#10) | Index and reference tests in sufficient detail to allow replication | 3-6 |
| Test methods | [#11](https://www.goodreports.org/reporting-checklists/stard/info/#11) | Rationale for choosing the reference standard (if alternatives exist) | 3-6 |
| Test methods | [#12](https://www.goodreports.org/reporting-checklists/stard/info/#12) | Definition of and rationale for test positivity cut-offs or result categories of the index and reference tests, distinguishing pre-specified from exploratory |  |
| 3-6 |  |  |  |
| Test methods | [#13](https://www.goodreports.org/reporting-checklists/stard/info/#13) | Whether clinical information and reference standard results were available to the performers / readers of the index test; Whether clinical information and index test results were available to the assessors of the reference standard |  |
| 3-6 |  |  |  |
| Analysis | [#14](https://www.goodreports.org/reporting-checklists/stard/info/#14) | Methods for estimating or comparing measures of diagnostic accuracy | 3-6 |
| Analysis | [#15](https://www.goodreports.org/reporting-checklists/stard/info/#15) | How indeterminate index test or reference standard results were handled | 3-6 |
| Analysis | [#16](https://www.goodreports.org/reporting-checklists/stard/info/#16) | How missing data on the index test and reference standard were handled | 3-6 |
| Analysis | [#17](https://www.goodreports.org/reporting-checklists/stard/info/#17) | Any analyses of variability in diagnostic accuracy, distinguishing pre-specified from exploratory | 3-6 |
| Analysis | [#18](https://www.goodreports.org/reporting-checklists/stard/info/#18) | Intended sample size and how it was determined | 3-6 |
| **Results** |  |  |  |
| Participants | [#19](https://www.goodreports.org/reporting-checklists/stard/info/#19) | Flow of participants, using a diagram | n/a |
| Participants | [#20](https://www.goodreports.org/reporting-checklists/stard/info/#20) | Baseline demographic and clinical characteristics of participants | 6-9 |
| Participants | [#21](https://www.goodreports.org/reporting-checklists/stard/info/#21) | Distribution of severity of disease in those with the target condition, and distribution of alternative diagnoses in those without the target condition | 6-9 |
| Participants | [#22](https://www.goodreports.org/reporting-checklists/stard/info/#22) | Time interval and any clinical interventions between index test and reference standard | 6-9 |
| Test results | [#23](https://www.goodreports.org/reporting-checklists/stard/info/#23) | Cross tabulation of the index test results (or their distribution) by the results of the reference standard |  |
| 6-9 |  |  |  |
| Test results | [#24](https://www.goodreports.org/reporting-checklists/stard/info/#24) | Estimates of diagnostic accuracy and their precision (such as 95% confidence intervals) | 6-9 |
| Test results | [#25](https://www.goodreports.org/reporting-checklists/stard/info/#25) | Any adverse events from performing the index test or the reference standard | 6-9 |
| **Discussion** |  |  |  |
| None | [#26](https://www.goodreports.org/reporting-checklists/stard/info/#26) | Study limitations, including sources of potential bias, statistical uncertainty, and generalisability | 9-12 |
| None | [#27](https://www.goodreports.org/reporting-checklists/stard/info/#27) | Implications for practice, including the intended use and clinical role of the index test | 9-12 |
| **Other information** |  |  |  |
| None | [#28](https://www.goodreports.org/reporting-checklists/stard/info/#28) | Registration number and name of registry | 13 |
| None | [#29](https://www.goodreports.org/reporting-checklists/stard/info/#29) | Where the full study protocol can be accessed | 3-6 |
| None | [#30](https://www.goodreports.org/reporting-checklists/stard/info/#30) | Sources of funding and other support; role of funders | 12-13 |

The STARD checklist is distributed under the terms of the Creative Commons Attribution License CC-BY. This checklist was completed on 01. October 2025 using <https://www.goodreports.org/>, a tool made by the [EQUATOR Network](https://www.equator-network.org) in collaboration with [Penelope.ai](https://www.penelope.ai)
